# Effects of high-dose glucose oxidase on broiler growth performance, antioxidant function, and intestinal microbiota in broilers

**DOI:** 10.3389/fmicb.2024.1439481

**Published:** 2024-10-28

**Authors:** Zipeng Jiang, Zhiyi Huang, Hongfang Du, Yangyuan Li, Min Wang, Dandie Chen, Jingyi Lu, Ge Liu, Liang Mei, Yuqi Li, Weifan Liang, Bo Yang, Yuguang Guo

**Affiliations:** ^1^Guangdong VTR Bio-tech Co., Ltd., Zhuhai, China; ^2^South China University of Technology, School of Biology and Biological Engineering, Guangzhou, China

**Keywords:** glucose oxidase, ultra-high doses, broilers, antioxidant ability, gut microbiota

## Abstract

Glucose oxidase (GOD) has been investigated as a potential additive for enhancing intestinal health and growth performance in poultry. However, limited research exists on the effects of ultra-high doses of GOD in practical poultry production. This study aimed to investigate the impact of high dietary GOD levels on broiler growth performance, antioxidant capacity, and intestinal microbiota. A total of 400 healthy, 1-day-old, slow-growing broiler chickens were randomly assigned to four treatment groups. The control group was fed a standard basal diet, while the other groups (G1, G2, and G3) were fed the basal diet supplemented with 4 U/g, 20 U/g, and 100 U/g of VTR GOD, respectively. The results showed that a dose of 100 U/g GOD significantly improved the final body weight and average daily feed intake (ADFI) (*p* < 0.05). Additionally, the G3 group exhibited a marked increase in glutathione peroxidase (GSH-Px) activity (*p* < 0.05), reflecting enhanced antioxidant function. Gut morphology remained intact across all groups, indicating no adverse effects on intestinal barrier integrity. Microbiota analysis revealed significant increases (*p* < 0.05) in Firmicutes and Verrucomicrobiota abundance at the phylum level in the GOD-supplemented groups. Moreover, GOD treatments significantly increased the abundance of *Faecalibacterium*, *Mucispirllum*, and *CHKCI001* at the genus level. Metabolic function predictions suggested that high-dose GOD supplementation enriched carbohydrate metabolism, particularly starch and sucrose metabolism. Correlation analysis indicated that *Faecalibacterium* and *CHCKI001* were two bacteria strongly influenced by GOD supplementation and were associated with enhanced growth performance and improved gut health. In conclusion, high-dose GOD supplementation had no adverse effects and demonstrated significant benefits, promoting both growth performance and gut health in broilers.

## 1 Introduction

Glucose Oxidase (GOD) is an aerobic dehydrogenase enzyme that uses a non-covalently bound coenzyme, flavin adenine dinucleotide (FAD). As an oxidoreductase, the flavoprotein catalyzes the oxidation of *β*-D-glucose into D-glucono-*δ*-lactone and hydrogen peroxide (H_2_O_2_), with molecular oxygen serving as the electron acceptor (Bankar et al., 2009). Due to its catalytic properties, GOD has gained significant commercial value and is widely used in various industries, including food processing, medical diagnostics, oral hygiene products, the chemical industry, and biotechnology (Mano, 2019; Tu et al., 2019; Zhao et al., 2017).

GOD plays a crucial role in neutralizing free oxygen radicals (Ding et al., 2022; Zhang et al., 2020). It catalyzes the oxidation of glucose to produce gluconolactone, using molecular oxygen as an electron acceptor (Yue Wang et al., 2023), which helps reduce oxidative tissue damage, preserve health, and promote growth. Due to these beneficial properties, GOD is considered an effective alternative to antibiotics (Liang et al., 2023; Zhao et al., 2023). GOD has emerged as a novel and promising feed additive in animal husbandry, with the majority of studies focusing on its effects as a feed additive aimed at maintaining animal health (Sun et al., 2022; Wang et al., 2018), enhancing growth performance (Tang et al., 2016; Zhao et al., 2021), and preventing pathogens (Liu et al., 2020; Wang et al., 2022; Wang et al., 2020) or mycotoxin infection (Qu and Liu, 2021; Gao et al., 2022; Zhao et al., 2022). Typically, 0–4 U/g of GOD is added to feed as an efficient and environmentally friendly additive (Wang et al., 2018; Tang et al., 2016; Wang et al., 2022; Zhao et al., 2022; Meng et al., 2021; Wang et al., 2022). One study found that 12 U/g of GOD could replace antibiotics and improve broiler growth performance (Zhao et al., 2023), while another used 100 U/g of GOD to improve the growth performance of pigs (Dang et al., 2021). However, the optimal dosage of GOD and the impact of excessively high doses on broiler production remain underexplored. Many users focus on the effects of adding lower concentrations of GOD to achieve practical production without fully considering the maximum effective limit at which GOD can exert its benefits. Therefore, this study experimentally investigated the effects of several high concentrations of GOD on broilers.

Growth performance is critical to all animal farming operations as it directly impacts economic returns. The gut microbiota plays a vital role in animal health, significantly influencing nutrition, digestion, absorption, and immune function (Li et al., 2023; Novoa Rama et al., 2023; Shi et al., 2019). During its catalytic process, GOD generates hydrogen peroxide, which has led some researchers to hypothesize that it might be detrimental to gut health. However, there is currently a lack of direct evidence to support the notion that GOD could negatively affect intestinal health or growth performance in animals. Furthermore, the optimal maximum additive amount of GOD has not been clearly established, limiting its potential and value in practical applications. Therefore, it is essential to conduct further animal experiments to determine whether high doses of GOD affect gut microbiota, antioxidant status, or growth performance, and to confirm its safety and efficacy.

In this study, based on our previous results, we aimed to investigate whether supplementing broiler chickens with high doses of GOD could positively impact growth performance, antioxidant status, and gut microbiota. This was accomplished through a series of animal experiments and various analytical techniques.

## 2 Materials and methods

All experimental procedures were approved by the Institutional Animal Care and Use Committee of Guangdong VTR Biotech and were conducted in strict accordance with the guidelines established by the National Institute of Animal Health.

### 2.1 Animals experimental design and diets

**Figure 1:**
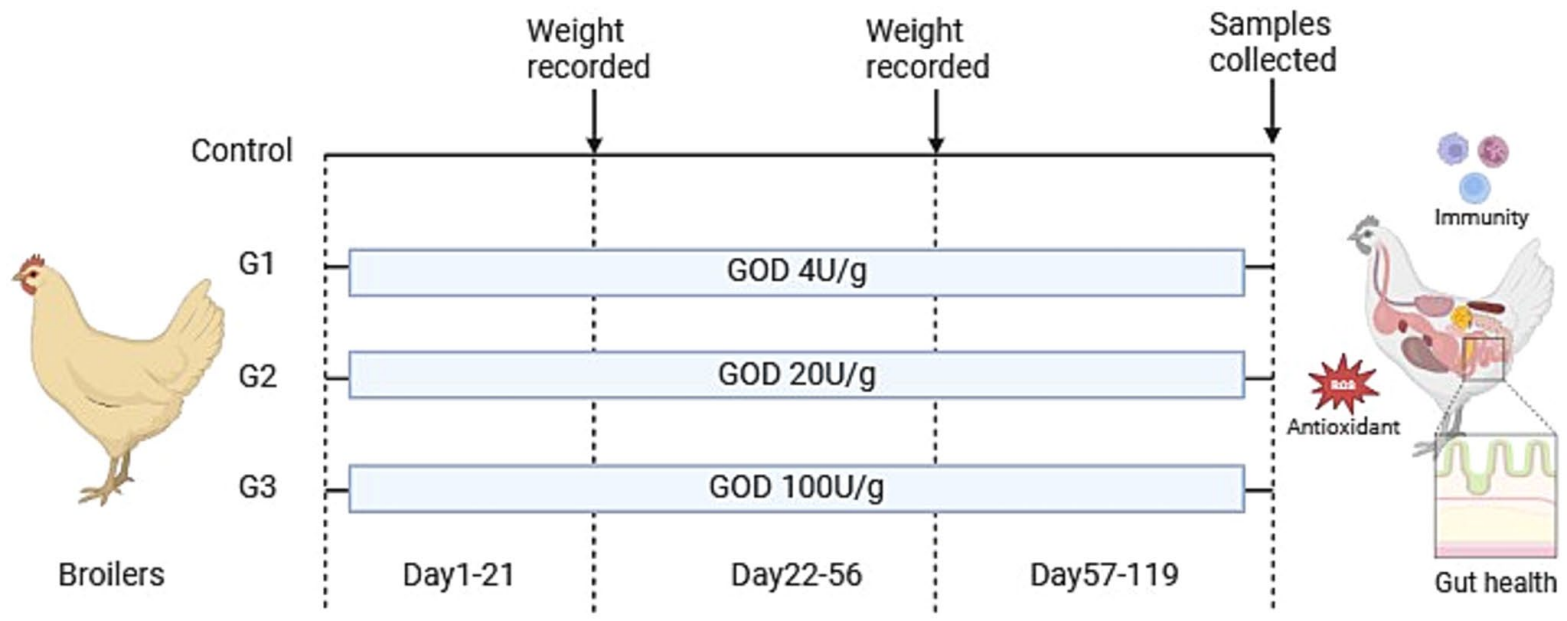
Experimental design and scheme of the animal treatments. (Created in BiorRender.com).

A total of 400 healthy one-day-old slow-growing broiler chickens (female chicks, purchased from Guangzhou Shunxin Agriculture and Animal Husbandry) were randomly assigned into four treatment groups, with 10 replicates per group and 10 broilers per replicate. The control was fed a basal diet, while G1, G2, and G3 were supplemented with 4 U/g, 20 U/g, and 100 U/g of VTR GOD in the basal diet, respectively (Figure 1).

The experiment was conducted in three stages: 1–21 days, 22–56 days, and 57–119 days of age. At the beginning and end of the experiment, the fasting weight of the broilers was measured per replicate group. The trial took place at the animal testing facility of Guangdong VTR Biotechnology Co., Ltd., where the broilers were housed in cages and managed under standard production practices, with free access to feed and water.

For the starter phase (10 broilers per cage), cage dimensions were 60 cm in length, 66 cm in width, and 44 cm in height. For the grower and finisher phases (two broilers per cage), cage dimensions length 43 cm in length, 40 cm in width, and 42 cm in height. Temperature control was maintained using infrared lamps and fans, with the first week’s temperature set at 31–33°C, the second week at 29.5°C, and a gradual reduction starting in the third week. Relative humidity was maintained at 60–65%, and the flock was vaccinated according to a routine immunization schedule.

The immunization procedures were as follows:

- 1-day old: Neck subcutaneous injection of Marek’s vaccine
- 7 days old: Newcastle Disease IV, branch 120, double vaccine drops in the nose and eyes 14 days old: Infectious bursa vaccine drops in the nose or eyes
- 21 days old: Second drinking water immunization with the infectious bursa vaccine
- 28 days old: Second drinking water immunization with Newcastle Disease IV, branch 120, double vaccine

The ingredient composition and calculated nutrient content of the experimental diets are shown in Supplementary Tables S1, S2. Throughout the experiment, daily feed intake, remaining feed, and mortality were meticulously recorded. At the beginning and end of the trial, the fasting weight of the broilers was measured for each replicate group to calculate key performance indicators, including average daily gain (ADG), average daily feed intake (ADFI), feed conversion ratio (FCR), and survival rate.

### 2.2 Sample collection

At the end of the experiment, six broilers from each group were randomly selected for sampling and euthanized using CO_2_. Serum was then obtained by centrifuging the blood at 3,000 g for 10 min at 4°C. After exsanguination, mid-segments (approximately 1 cm) of the jejunum from one broiler per replicate were collected and fixed in a 10% paraformaldehyde solution for morphology analysis. Additionally, jejunal segments were collected for mRNA or protein determination. Cecal contents were collected and stored at −80°C for microbiota composition analysis.

### 2.3 Intestinal morphology

After fixation in 4% paraformaldehyde, gut samples were stained with hematoxylin and eosin (H&E). Intestinal morphology was analyzed following previously established methods (Hao et al., 2021). The measurements included villus height, intestinal crypt depth, and the villus-to-crypt (V/C) ratio to assess intestinal structure.

### 2.4 Serum antioxidant ability

Serum antioxidant indices, including superoxide dismutase (SOD), glutathione peroxidase (GSH-Px), and the damage index malondialdehyde (MDA), were measured using ELISA kits (provided by Jiangsu Meibiao Biotechnology Co., Ltd., China).

### 2.5 Quantitative real-time PCR

Total RNA was extracted using an RNA kit (Invitrogen, United States). RNA concentration was measured using the NanoDrop 2000 spectrophotometer. Quantitative real-time PCR was then performed using a CFX96 Real-Time System (Bio-Rad Laboratories). Primer sequences used for qPCR are shown in Supplementary Table S3. The reference gene *GAPDH* was used as a housekeeping gene. Relative mRNA expression levels were calculated using the 2^−ΔΔCt^ method.

### 2.6 Microbial analysis

Genomic DNA from the cecal contents was extracted using the PowerSoil DNA isolation kit (MoBio Laboratories Inc., USA) following the manufacturer’s protocol. Subsequently, the V3-V4 region of the bacterial 16S rRNA gene (primers 515F: ACTCCTACGGGAGGCAGCAG, 806R: GGACTACHVGGGT-WTCTAAT) was amplified through PCR. Sequencing was conducted using the Illumina Hiseq2500 platform, which was conducted according to previous studies (Su et al., 2022; Jiang et al., 2021). The clean sequences were classified into identical amplicon sequence variants (ASVs). Chao, Simpson, Shannon, and Good’s coverage indices were calculated using the QIIME2 (http://qiime2.org/) pipeline, with DADA2 (Version 1.8) used for denoising sequences (Edgar and Flyvbjerg, 2015). UniFrac-based principal coordinate analysis (PCoA) was conducted to visualize the microbial community differences. Linear discriminant analysis effect size (LEfSe) was used to identify the main differentially abundant genera. Tax4Fun was used to predict the functional profile of the gut microbiota. The sequence data were deposited in the Sequence Read Archive (SRA) under accession number PRJNA1116544.

### 2.7 Spearman correlation analysis of gut bacteriome, growth performance, antioxidant parameters, tight junction protein mRNA, and V/C

We conducted a Spearman correlation analysis to identify key microorganisms associated with the potential gut health benefits of GOD supplementation. This analysis assessed the relationship between changes in gut microbiota composition, growth performance, antioxidant markers, mRNA expression levels of tight junction proteins, and the V/C ratio. Spearman’s correlation was conducted using R (version 4.2), considering an absolute value of correlation coefficients >0.45 and a *p-*value of <0.05 as statistically significant.

### 2.8 Statistical analysis

Data analysis was conducted using SPSS 22.0 software (IBM Company, Armonk, NY). Significant differences between groups were determined using one-way ANOVA followed by Duncan’s multiple range test, with a *p-*value of < 0.05 considered statistically significant. The results are expressed as mean ± SD. In addition, data visualization and graphing were conducted using GraphPad Prism version 9.0 (San Diego, CA, USA) and R version 4.2.

## 3 Results

### 3.1 Growth performance of broilers

Table 1 shows that incorporating GOD into the diet at levels of 4 U/g and 20 U/g had no significant impact on the broilers’ daily feed intake throughout each week (*p* < 0.05). In contrast, supplementation with 100 U/g GOD significantly increased weekly daily feed intake from weeks 13 to 17 in the G3 group. Table 2 shows that, compared to the control group, GOD supplementation at various doses enhanced the growth performance of broilers, leading to an increase in ADG and a reduction in FCR during days 1–21. The improvement in growth appears to be directly proportional to the amount of GOD added.

**Table 1:** Weekly daily feed intake (g/d).

Treatment^1^	Control	G1	G2	G3	*p*- value
Week 1	14.78 ± 0.53	15.24 ± 0.40	15.46 ± 0.80	15.03 ± 0.60	0.121
Week 2	18.31 ± 0.26	18.14 ± 0.83	18.17 ± 0.69	18.22 ± 0.61	0.649
Week 3	34.49 ± 2.27	34.50 ± 1.17	34.49 ± 1.17	34.15 ± 2.55	0.953
Week 4	45.24 ± 1.33	45.69 ± 1.41	45.73 ± 1.35	45.96 ± 1.78	0.910
Week 5	63.19 ± 0.85	63.21 ± 1.12	62.79 ± 0.85	63.34 ± 1.20	0.840
Week 6	68.59 ± 1.40	68.45 ± 1.58	68.76 ± 1.10	69.00 ± 1.33	0.932
Week 7	72.47 ± 1.71^c^	75.65 ± 2.66^ab^	74.21 ± 1.75^bc^	76.49 ± 2.35^a^	0.000
Week 8	78.77 ± 2.70^b^	81.95 ± 3.30^ab^	83.57 ± 1.71^a^	83.79 ± 3.50^a^	0.005
Week 9	88.37 ± 4.48	91.69 ± 3.78	89.54 ± 3.61	90.66 ± 3.61	0.208
Week 10	95.81 ± 3.23^b^	99.51 ± 6.60^ab^	95.67 ± 2.93^b^	101.30 ± 2.56^a^	0.020
Week 11	101.23 ± 1.87	104.37 ± 8.09	100.86 ± 3.23	101.05 ± 3.91	0.542
Week 12	96.91 ± 3.75^a^	98.46 ± 3.40^a^	92.86 ± 5.73^b^	96.98 ± 3.44^a^	0.003
Week 13	114.37 ± 11.24	111.46 ± 8.93	110.05 ± 8.39	116.03 ± 7.38	0.233
Week 14	103.58 ± 7.58^b^	104.74 ± 4.68^b^	105.02 ± 7.34^b^	112.68 ± 5.96^a^	0.005
Week 15	120.24 ± 11.07	121.75 ± 7.28	118.40 ± 6.28	124.33 ± 6.41	0.520
Week 16	119.40 ± 6.85^b^	120.92 ± 8.06^b^	123.02 ± 7.76^ab^	127.65 ± 7.50^a^	0.042
Week 17	118.83 ± 6.77^b^	120.83 ± 5.95^b^	123.67 ± 5.62^b^	131.07 ± 6.21^a^	0.000

^1^Control, no additives; G1-3: GOD 4 U/g, 20 U/g, and 100 U/g. ^a,b,c^Values within a row with different letters differ significantly (*p* < 0.05).

**Table 2:** Effect of GOD on the Growth performance of Broilers in starter to finisher Phases.

Treatment^1^	Control	G1	G2	G3	*p*- value
Starter phase (d0-21)					
ADG (g/d)	11.39 ± 0.92	11.40 ± 0.66	11.61 ± 0.64	11.78 ± 0.65	0.523
ADFI (g/d)	22.53 ± 0.66	22.63 ± 0.41	22.70 ± 0.57	22.47 ± 0.75	0.885
FCR	1.99 ± 0.17	1.99 ± 0.09	1.96 ± 0.11	1.91 ± 0.10	0.357
Survival rate (%)	100	100	100	100	1
Grower phase (d22-56)					
ADG (g/d)	23.50 ± 0.97	22.74 ± 0.83	22.74 ± 0.44	23.29 ± 0.80	0.246
ADFI (g/d)	65.65 ± 0.90^b^	66.99 ± 1.01^a^	67.01 ± 0.52^a^	67.72 ± 0.63^a^	0
FCR	2.80 ± 0.12^b^	2.95 ± 0.09^a^	2.95 ± 0.06^a^	2.91 ± 0.10^a^	0.036
Survival rate (%)	100	100	100	100	1
Finisher phase (d57-119)					
ADG (g/d)	12.92 ± 1.68	13.93 ± 1.73	14.04 ± 1.36	14.94 ± 1.78	0.119
ADFI (g/d)	112.22 ± 5.41^b^	113.03 ± 4.08^b^	112.17 ± 4.61^b^	118.12 ± 3.73^a^	0.003
FCR	8.80 ± 1.01	8.22 ± 1.04	8.06 ± 0.82	8.02 ± 1.05	0.462
Survival rate (%)	100	98.90 ± 3.48	99.00 ± 3.16	97.90 ± 4.43	0.61
Starter to finisher phase (d0-119)					
Initial weight (g)	30.50 ± 0.33	30.45 ± 0.28	30.45 ± 0.28	30.40 ± 0.39	0.967
Final weight (g)	2270.49 ± 66.84^b^	2274.31 ± 41.12^b^	2291.06 ± 48.45^b^	2348.53 ± 85.29^a^	0.0499
ADG (g/d)	18.82 ± 0.56	18.86 ± 0.34	19.00 ± 0.41	19.48 ± 0.72	0.0502
ADFI (g/d)	79.68 ± 1.52^b^	80.89 ± 2.07^b^	80.13 ± 1.82^b^	82.77 ± 1.57^a^	0.001
FCR	4.24 ± 0.13	4.29 ± 0.15	4.22 ± 0.11	4.25 ± 0.17	0.856
Survival rate (%)	100	98.90 ± 3.48	99.00 ± 3.16	97.90 ± 4.43	0.61

ADG, Average daily gain; ADFI, average daily feed intake; FCR, feed conversion ratio. ^1^Control, no additives; G1-3: GOD 4 U/g, 20 U/g, and 100 U/g. ^a,b,c^Values within a row with different letters differ significantly (*p* < 0.05).

During days 22–56, compared to the control group, ADFI increased significantly (*p* < 0.05) in the G1, G2, and G3 groups, although this did not result in a corresponding improvement in ADG or a reduction in FCR. In the Finisher phase (days 57–119), GOD improved final body weight, increased the ADFI of the yellow-feathered broilers, and reduced FCR. The 100 U/g GOD was the most effective, significantly improving ADFI (*p* < 0.05), increasing ADG by 15.63%, and reducing FCR by 8.86%.

Throughout the entire experimental period, an increasing trend was observed in the G1, G2, and G3 groups for final weight, ADG, and ADFI. The 100 U/g GOD dose significantly increased the final weight by 3.44% (*p* < 0.05) and ADFI by 3.88% (*p* < 0.05). The effect of GOD on enhancing growth performance appeared to be directly proportional to the amount of GOD added.

### 3.2 GOD improved antioxidant function

The results for the serum antioxidant ability are presented in Table 3. Compared to the control group, SOD levels showed a gradual increase in the G1, G2, and G3 groups, though the differences were not statistically significant among these groups. Similarly, the MDA concentration in the serum decreased in the G2 and G3 groups, but no significant reduction was observed between them. Notably, GSH-Px levels significantly increased in the G3 group (*p* < 0.05).

**Table 3:** Effect of GOD on the antioxidant ability of broilers (Serum).

Treatment^1^	Control	G1	G2	G3	*p*- value
SOD (U/mL)	444.61 ± 160.73	495.15 ± 67.17	574.58 ± 77.82	526.09 ± 42.99	0.468
MDA (nmol/mL)	3.78 ± 1.65	3.78 ± 0.19	3.22 ± 1.17	2.67 ± 0.67	0.552
GSH-Px (U)	2371.43 ± 318.16^b^	2447.62 ± 280.43^b^	3419.05 ± 847.73^ab^	4361.90 ± 554.27^a^	0.007

^1^Control, no additives; G1-3: GOD 4 U/g, 20 U/g, and 100 U/g. ^a,b,c^Values within a row with different letters differ significantly (*p* < 0.05).

### 3.3 GOD improved intestinal barrier function

**Figure 2:**
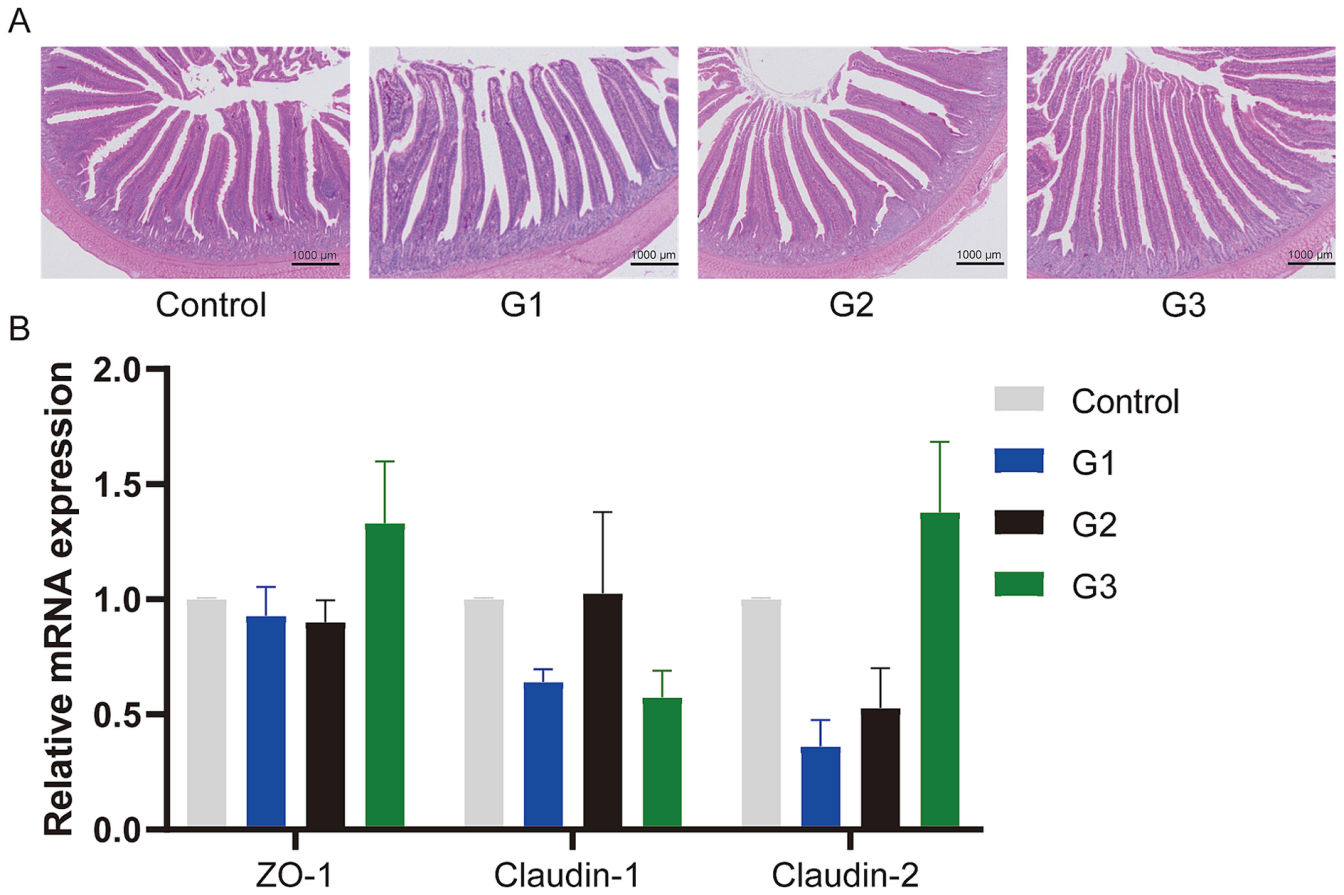
GOD enhanced the gut barrier function. (A) Jejunum tissue stained with hematoxylin and eosin (H&E) (microscope magnification 20×, bars = 1,000 μm). (B) The relative mRNA expression of Jejunum tissue. The results are presented as mean ± SD (*n* = 6).

Figure 2 shows the jejunum villus and crypt morphology analysis. Compared to the control group, the GOD addition groups exhibited continuous brush borders and intact villi, suggesting that the gut structure and gut physical barrier were normal. Specifically, Table 4 presents the villus height, crypt depth, and the V/C ratio. The G1 group showed that the GOD significantly increased the villus height and the V/C ratio (*p* < 0.05) in the duodenum segment. In the jejunum and ileum sections, compared to the control group, the GOD groups showed an increase in the villus height and the V/C ratio in the ileum and villus height in the jejunum. The G2 group exhibited a noticeable reduction in crypt depth in the jejunum.

**Table 4:** Effect of GOD on intestinal morphology of broilers (Jejunum).

Treatment^1^	Control	G1	G2	G3
Villus Height (μm)				
Duodenum	1447.02 ± 157.89^b^	1985.97 ± 195.10^a^	1624.01 ± 199.22^b^	1570.92 ± 171.09^b^
Jejunum	1292.42 ± 241.35	1480.32 ± 185.52	1394.97 ± 257.50	1561.05 ± 61.98
Ileum	1083.59 ± 86.20	1146.88 ± 62.20	1153.21 ± 232.83	1333.68 ± 57.82
Intestinal Crypt (μm)				
Duodenum	268.19 ± 67.49	283.50 ± 5.97	247.52 ± 36.04	325.12 ± 69.43
Jejunum	197.13 ± 68.01	197.33 ± 10.41	187.09 ± 54.72	253.48 ± 80.74
Ileum	153.99 ± 9.66	156.40 ± 16.55	157.81 ± 17.61	161.18 ± 41.95
V/C				
Duodenum	5.93 ± 0.92^b^	7.90 ± 1.25^a^	6.85 ± 0.20^ab^	5.21 ± 1.10^b^
Jejunum	7.41 ± 1.19	7.93 ± 1.19	8.16 ± 1.15	6.85 ± 2.14
Ileum	7.41 ± 0.22	8.36 ± 0.17	8.05 ± 1.18	9.36 ± 1.96

^1^Control, no additives; G1-3: GOD 4 U/g, 20 U/g, and 100 U/g. ^a,b,c^Values within a row with different letters differ significantly (*p* < 0.05).

### 3.4 GOD improved gut microbiota

Microbial analysis was conducted using 16 s rRNA sequencing. Table 5 shows that Good’s coverage for all samples was 0.99, indicating that the sequencing depth was sufficient for further analysis. The total number of ASVs and Chao1 increased by the GOD treatment. However, the number of observed ASVs, Chao1 index, and Shannon index had no differences among the four groups. The Simpson index decreased significantly (*p* < 0.05) in the G3 group compared to the other groups.

**Table 5:** Effect of GOD on the α-diversity of intestinal microbiota in broilers (Cecal contents).

Item	Control	G1	G2	G3
Good’s coverage	0.99 ± 0	0.99 ± 0	0.99 ± 0	0.99 ± 0
ASVs	356.0 ± 17.64	382.66 ± 23.06	443.50 ± 39.17	423.30 ± 34.43
Chao 1	400.92 ± 21.34	432,32 ± 31.41	483.72 ± 39.95	491.96 ± 33.24
Shannon	5.93 ± 0.13	5.99 ± 0.08	6.14 ± 0.20	5.74 ± 0.19
Simpson	0.96 ± 0.00 ^a^	0.96 ± 0.00 ^a^	0.96 ± 0.00 ^a^	0.94 ± 0.00 ^b^

^a,b^Values within a row with different letters differ significantly (*p* < 0.05).

**Figure 3:**
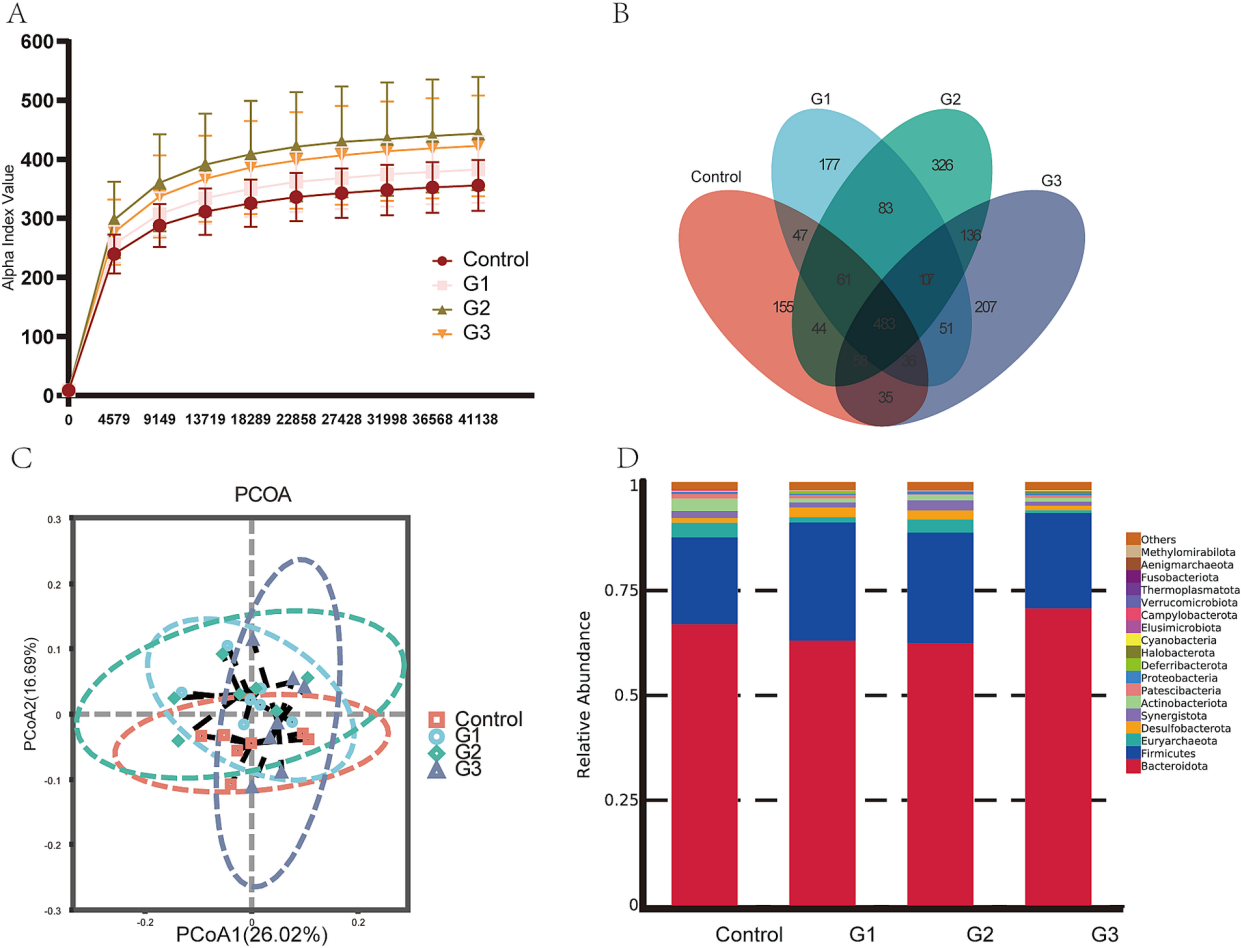
GOD influenced the gut microbiota in broilers (cecal contents) (*n* = 6). (A) Observed ASV line chart. (B) Venn diagram. (C) Principal coordinated analysis (PCoA). (D) Relative abundance bar chart in Phylum-level.

Figure 3A shows that the number of observed ASVs in the G2 group was higher (*p* < 0.05) than in other groups. Figure 3B shows that the 83 ASVs were shared by all treatments, and the G2 group had unique microbes (326). Furthermore, PCoA analysis in Figure 3C shows that bacterial community structure in GOD treatment groups was visibly separated from the control group, suggesting GOD treatment made a substantial impact on microbiota *β* diversity. The bar chart illustrates the compositions of microbiota at the phylum level in Figure 3D. The predominant phylum in the digest of the four groups was Firmicutes and Bacteroidetes. Compared to the control group, Firmicutes significantly increased in the GOD treatment groups (*p* < 0.05). The G3 group was distinguishable from the others because it had the highest relative abundance of Verrucomicrobiota, which could help the host maintain gut health. The relative abundance of Actinobacteriota was higher in the control and G2 groups than in the G1 and G3 groups.

**Figure 4:**
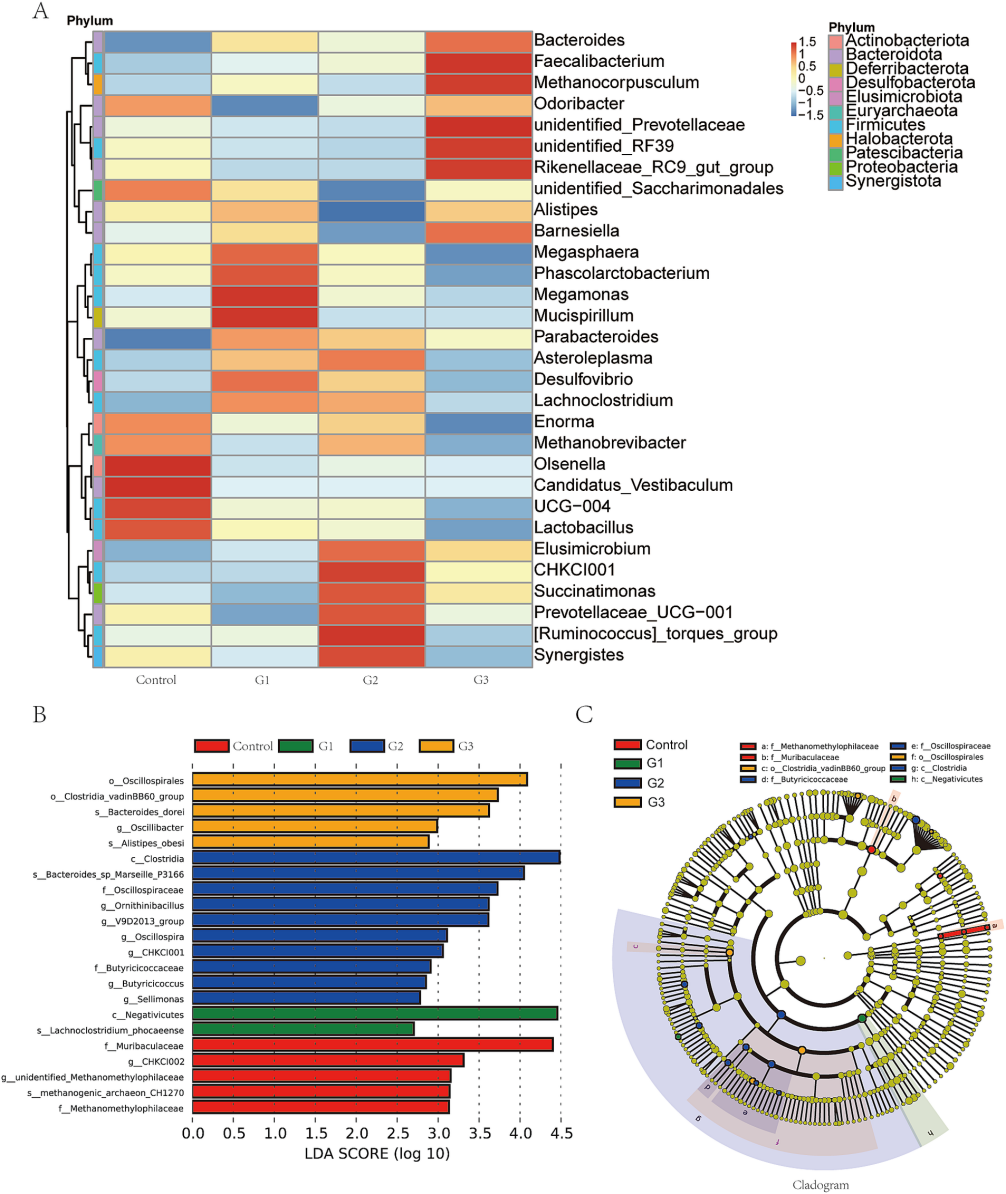
GOD altered the intestinal microbial community structure in broilers (cecal contents). (A) Heatmap in genus level. (B) Linear discriminant analysis LDA scores (> 2.5) were calculated for features at the amplicon sequence variance (ASV) level. Letters represented the taxonomy of the bacteria: p, phylum; c, class; o, order; f, family; g, genus. (C) Cladogram in genus level.

At the genus level, Figure 4A shows GOD treatment increased the abundance of *Bacteroides*, *Faecalibacterium*, *Methanocorpusculum*, *Megamonas*, *Mucispirllum*, and *CHKCI001* while reducing the abundance of *Olsenella*, *Enorma*, and *Candidatus Vestibaculum*. Figure 4B shows the significant differences in bacteria from the phylum to the species level. Notably, we found that the G2 group increased the abundance of Clostridia at class level, Oscillospiraceae, Butyricicoccaceae at family level, *Omithinibacillus*, *V9D2013 group*, *Oscillospia*, *CHCKI001*, *Butyricicoccus*, *Sellimonas* in genus level, and *Bacteroides* sp. *Marseille P3166* in species level. Figure 4C uses a cladogram to visualize the specific difference between the four groups. Red represented the control group, and green, blue, and orange represented the G1, G2, and G3 groups, respectively.

### 3.5 GOD improved gut microbiota metabolic function

**Figure 5:**
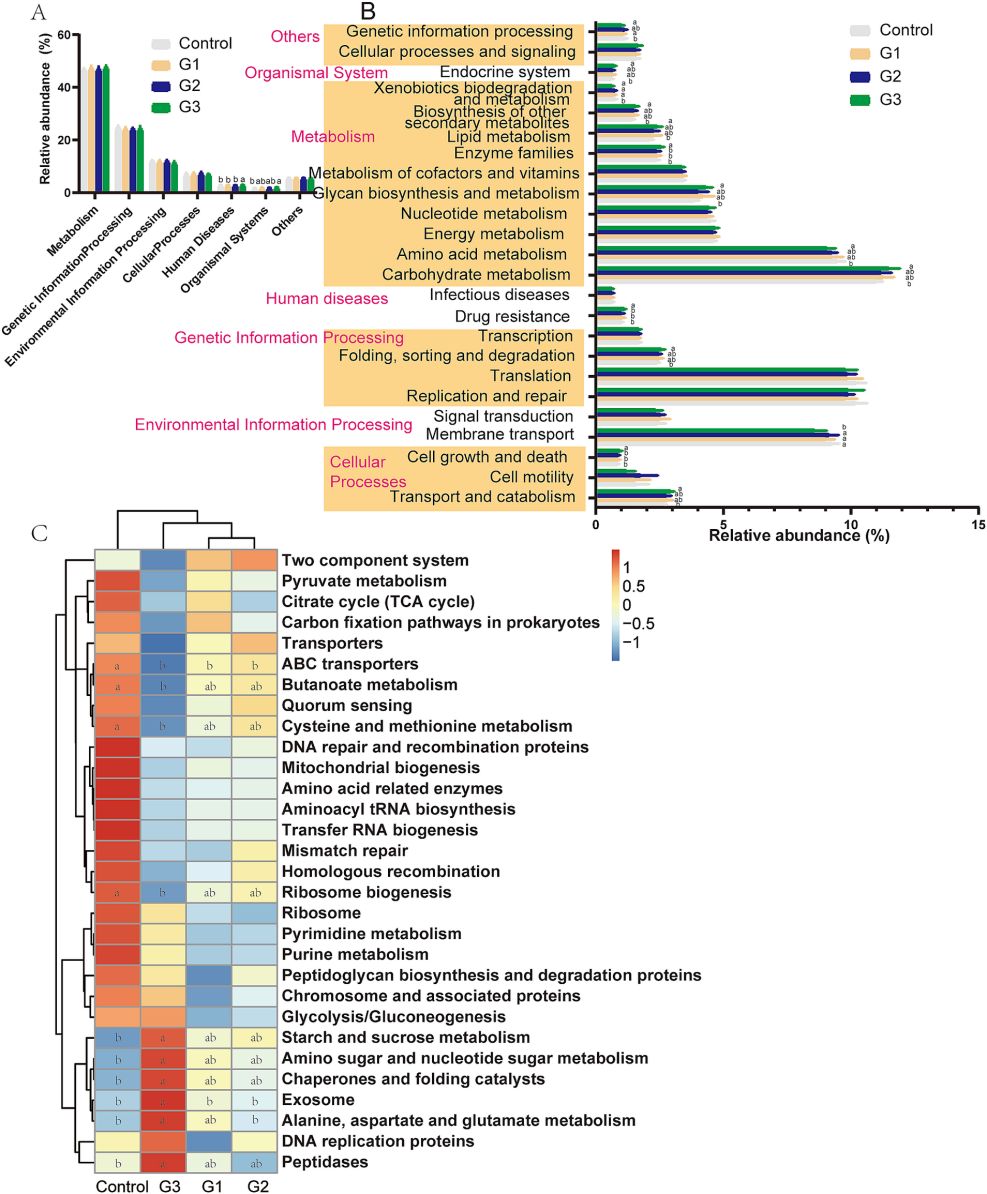
Dynamic bacterial functional profiles were analyzed by Tax4Fun (Cecal contents) (*n* = 6). (A) Metabolic pathways in level 1. (B) Metabolic pathways in level 2. (C) Functional predictions of the relative abundances of the top 30 metabolic functions (Level 3). a, b Means with a row with different superscripts significantly differ (*p* < 0.05).

The metabolic functions of the gut microbiota were predicted using the KEGG pathway database with the Tax4Fun tool. Figures 5A,B show that the cecal bacterial metabolic functions (Level 1 and Level 2) in the G3 group were enriched in cellular processes (transport and catabolism, cell growth and death), genetic information processing (folding, sorting, and degradation), metabolism (carbohydrate metabolism, amino acid metabolism, glycan biosynthesis, and metabolism, enzyme families, lipid metabolism, and biosynthesis of other secondary metabolites), and the organismal system (endocrine system).

The results of the predicted function at Level 3 (Figure 5C) further showed that the high doses of GOD treatment enriched pathways related to starch and sucrose metabolism, amino sugar and nucleotide sugar metabolism, chaperones and folding catalysts, exosome function, alanine, aspartate, and glutamate metabolism, and peptidases. However, GOD also led to a reduction in ABC transporters, butanoate metabolism, cysteine and methionine metabolism, and ribosome biogenesis metabolic function in the G3 group.

### 3.6 Spearman correlation analysis revealed the relationship between the microbiota and basic index

**Figure 6:**
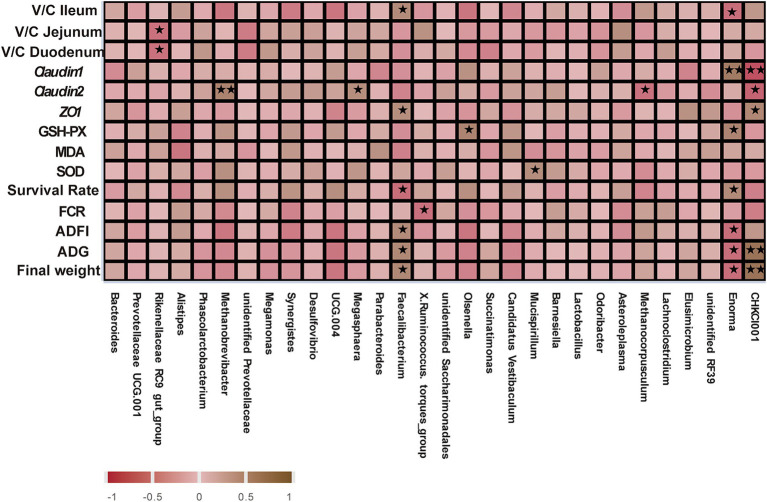
Spearman correlation analysis of gut bacteriomes (cecal contents), growth performance, antioxidant parameters, tight junction protein mRNA expression, and V/C. Significant correlation is represented by **0.001 < *p* < 0.01, *0.01 < *p* < 0.05, respectively. All the values contained six repetitions.

To identify the main gut microorganisms for GOD to improve gut health, spearman correlation analysis was conducted to study the relationship among differential gut microbiota, growth performance, antioxidant parameters, tight junction protein mRNA expression, and V/C ratio. Figure 6 shows that *Methanobrevibacter* (*p* < 0.01) and *Megasphaera* (*p* < 0.05) were positively correlated with *Claudin-2* expression, and these two bacteria were enriched in the G2 and G3 groups. Meanwhile, the *Faecalibacterium* was positively correlated with the final weight, ADG, ADFI, *ZO-1,* and V/C of the ileum (*p* < 0.05). *Mucispirillum,* which was enriched in the G2 group, was positively correlated with the SOD (*p* < 0.05). *Enorma* had a negative relationship with ADFI, ADG, V/C ratio of ileum, and final weight of broilers in the control group (*p* < 0.05). Moreover, the *CHCKI001,* which was enhanced in the G3 group, showed a positive relationship with the ADG and the final weight (*p* < 0.01) and maintained a positive correlation with *ZO-1* expression (*p* < 0.05). These findings suggest these microorganisms play a significant role in improving gut health and the overall performance of the animals.

## 4 Discussion

GOD catalyzes the oxidation of *β*-D-glucose into gluconic acid, using atomic oxygen as the electron acceptor. This process concurrently generates hydrogen peroxide, which can enhance intestinal health. Some studies suggest that when GOD enters the gut with feed, it consumes oxygen (Kundu et al., 2013), creating a relatively anaerobic environment in the gut that eliminates pathogens. This environment increases villus height, decreases crypt depth, and enhances the surface area available for digestion and absorption in the intestinal tract. Relevant studies have proven that GOD can inhibit the growth and spore production of *Fusarium solani* (Kriaa et al., 2015). Additionally, another study confirmed that GOD could completely eliminate mastitis pathogens, with the exception of *Ps. aeruginosa* (Sandholm et al., 2010). Furthermore, due to the reduction in pathogens, the secretion of diamine oxidase and D-lactate decreased, which subsequently alleviated inflammation in intestinal epithelial cells caused by pathogens (Liu et al., 2020; Zhao et al., 2022). The increased villi height-to-crypt depth ratio also contributed to improved intestinal epithelial cell proliferation. Evidence shows that the gene expression of tight junction proteins (ZO-1, claudin-1, and claudin-2) was enhanced by the addition of GOD (Teng et al., 2020). The physical barrier of the gut is crucial for maintaining intestinal health (Wang et al., 2024), and GOD effectively maintains the expression of tight junction proteins and protects the integrity of intestinal villi. These findings suggest that GOD has the potential to improve digestive and absorptive functions by enhancing gut health.

Additionally, GOD’s effectiveness at the macro level is reflected in the improved growth performance in animals. Numerous studies have reported that GOD treatment could increase growth performance, including ADG, ADFI, and the final weight. Zhao et al. found that the feed-to-gain ratio significantly decreased in the group supplied with 1,200 U/kg GOD (GOD1200) compared to the antibiotic group (Zhao et al., 2023). Wu et al. showed that the GOD-P treatment group, by modulating the intestinal microbiota, significantly increased ng ADG and ADFI in broiler chickens, improving meat quality at 21 and 42 days of age (Wu et al., 2020). Research has shown that the addition of 250 units of GOD per kilogram of feed can enhance weight gain in broiler chickens from 22 to 42 days of age, as well as increase the apparent ileal digestibility of certain amino acids (Meng et al., 2021). Wu et al. also found that broilers supplemented with GOD exhibited increased ADG, improved meat quality, and enhanced digestive capacity, with positive effects comparable to those observed in the group supplemented with antimicrobial growth promoters (AGPs) (Wu et al., 2019). However, some studies have reported no significant effects of GOD on growth performance in broilers. Meng et al. found that adding 500 or 1,000 units of GOD per kilogram of feed did not significantly affect ADG, ADFI, or other growth parameters (Meng et al., 2021). Similarly, Wang et al. reported that supplementing broiler diets with 75 U/kg of GOD did not have a significant impact on broilers’ growth performance (Wang et al., 2018).

Other studies also presented that GOD could prevent the *Clostridium perfringens*, *E.coli,* or mycotoxin infection. 200 U/kg GOD in the diet has been shown to improve the growth performance of ducks infected with 3 × 10^9^ CFU/mL *E.coli* O88 (Liu et al., 2020). GOD supplementation (150 U/kg) alleviated the decrease in the ADG and ADFI triggered by *Clostridium perfringens* infection (Zhao et al., 2022). Gao et al. discovered that exposure to Aflatoxin B1 (AFB1) and lipopolysaccharide (LPS) can lead to a reduction in the final body weight, ADG, and ADFI of broiler chickens, while supplementation with GOD or *Bacillus subtilis* has been shown to counteract the adverse effects of these toxins on the growth performance and FCR of broiler chickens (Gao et al., 2022). These studies demonstrate that appropriate doses of GOD can improve growth performance and prevent pathogen or mycotoxin infections in animals. However, they do not address the effects of excessive doses of GOD on animals. In the present study, we focused on growth performance and found that dietary supplementation with 100 U/g GOD significantly increased the final weight, ADG, and ADFI in broilers, a finding that has not been previously reported. Similarly, the G2 group with GOD supplementation of 25 U/g also showed the potential to increase broilers’ growth performance.

Because of their high lipid content, broilers are susceptible to the generation of reactive oxygen species (ROS) (Bai et al., 2017). To counteract the excess ROS, two key antioxidant enzymes, SOD and GSH-Px, were utilized to neutralize the harmful effects of these reactive molecules (Hasko et al., 2004). MDA was a metabolite from lipid peroxidation and a biomarker for oxidative stress (Cordiano et al., 2023). One study reported that 1,200 U/kg of GOD significantly increased SOD activity and decreased MDA levels (Zhao et al., 2023). Another study confirmed the same results, stating that GOD treatment significantly increased the activity of GSH-Px in the jejunal mucosa (Wang et al., 2022). In this study, we demonstrated that GOD could effectively alleviate oxidative stress induced by ROS, and 100 U/g GOD significantly increased GSH-Px concentration in broilers, confirming that high doses of GOD had positive effects on animals.

Enhancements in the nutritional quality of diets can significantly impact intestinal morphology and the functionality of the intestinal barrier (Schoultz and Keita, 2020). Intestinal morphology is primarily reflected in the villus height, crypt depth, and microvilli integrity. The intestinal mucosal barrier serves as the first line of defense against pathogens, with the core tight junction proteins complex, including ZO proteins (Zonula occludens) and claudin, playing an important role in maintaining this barrier’s function (Chelakkot et al., 2018; König et al., 2016). *Enterotoxigenic Escherichia coli* (ETEC) challenges have been shown to increase serum alanine transaminase activity, leading to intestinal morphological damage and inflammation. However, GOD has been found to counteract these harmful effects (Wang et al., 2022). Meng et al. reported that the addition of 250 U/kg GOD improved intestinal morphology by increasing villus height and enhancing the villus height to crypt depth ratio, both key indicators of intestinal health (Meng et al., 2021). Regarding tight junction protein mRNA expression, Wang et al. showed that 75 U/kg GOD increased the expression of tight junction protein genes (Wang et al., 2018). Similarly, Liu et al. found that 200 U/kg GOD increased the expression of *ZO-1*, *Claudin-1,* and *Claudin-2* genes (Liu et al., 2020). Our current study corroborates these findings, showing that GOD helps maintain intestinal integrity by increasing villus height, reducing crypt depth, and promoting dense and intact microvilli while also upregulating the expression of tight junction protein-related genes. These results suggest that high doses of GOD do not cause adverse side effects in broilers and contribute to maintaining intestinal health. These studies demonstrate that GOD is a key factor in maintaining gut health. The main reasons can be summarized as follows: First, GOD consumes oxygen, creating an anaerobic gut environment that reduces the prevalence of pathogens, such as *Escherichia coli O88* and *Clostridium perfringens*, thereby preserving the gut’s biological barrier function (Liu et al., 2020; Wang et al., 2022; Zhao et al., 2022). Second, GOD enhances the physical barrier of the gut by enhancing the integrity of intestinal villi, stimulating intestinal cell proliferation, and increasing the expression of tight junction proteins (Meng et al., 2021; Teng et al., 2020). Third, GOD supports immune function and reduces oxidative stress in intestinal cells by decreasing the release of inflammatory factors (IFN-*γ*, IL-1*β*, IL-6, and TNF-*α*) (Wang et al., 2018; Liu et al., 2020; Wang et al., 2022), promoting the secretion of anti-oxidative enzymes, including SOD, CAT, and GSH-Px, and reducing MDA concentrations, all of which contribute to the fundamental functions of the gut (Zhao et al., 2023; Qu and Liu, 2021). Inflammatory factors can cause significant intestinal damage, and maintaining a balance between pro-inflammatory and anti-inflammatory factors is crucial for the homeostasis of intestinal cells (Li et al., 2020; de et al., 2021; Wang et al., 2024). Enzymes such as SOD and GSH-Px are essential for removing excess ROS (Ko et al., 2004), and they have demonstrated significant benefits in animal models (Pamplona and Costantini, 2011).

Then, we show how GOD promotes gut microbial homeostasis. The cecal microbiota of broilers can influence the host’s health and productivity, as the gut microbiota plays an essential role in maintaining gut health. Therefore, analyzing the microbiota could help identify its core components (Stanley et al., 2014). Analysis of microbial community alpha (α) and beta (β) diversity revealed significant differences in the cecal microbiota between the moldy corn group and the 0.01% GOD groups (Qu and Liu, 2021). Alpha diversity (Chao1, Shannon, Simpson index) often represents the species diversity of intestinal microorganisms. In our study, using 100 U/g GOD improved the diversity of microbiota by reducing the Simpson index. PCoA analysis revealed differences in beta diversity among these groups. There was a significant difference in the GOD treatment group compared to the control group, which indicated that the high doses of GOD reshape the gut microbiota in broilers. Wu et al. revealed significant alterations in the abundance of the phylum Firmicutes, the families Ruminococcaceae and Rikenellaceae, and the genus *Faecalibacterium*, specifically the species *F. prausnitzii* (Wu et al., 2019). Wang et al. found that treatment with GOD significantly increased the relative abundance of the *Bacteroides* genus, which play a crucial role in the breakdown of complex carbohydrates in the gut (Wang et al., 2022). 250 U/kg GOD treatment resulted in elevated relative abundances of the phylum Firmicutes, known for their role in digestion and energy homeostasis, and the genus *Lactobacillus*. These beneficial bacteria contribute to gut health. In contrast, the relative abundance of *Escherichia-Shigella* decreased (Meng et al., 2021). Similarly, the relative abundance of the Firmicutes phylum, another major group of gut bacteria, was observed to increase with the present GOD treatment. The alteration in microbiota composition may impact gut health and digestive efficiency. In the group treated with the GOD, the ratio of Bacteroidetes to Firmicutes was higher compared to the control group (Kim et al., 2021). In the group raised on thick bedding material with added 200 U/kg of GOD, the relative abundance of the Firmicutes and Bacteroidetes phyla in the gut microbiota was significantly higher at 42 days of age (Zhao et al., 2021). These findings suggest that GOD can promote a balanced and diverse gut microbiota, which is essential for maintaining optimal gut health and enhancing digestive efficiency in broilers.

Furthermore, *Akkermansia muciniphila* is the predominant member in the Verrucomicrobiota, which proves that it had a strong positive correlation with gut health (Han and Zhuang, 2021), and Verrucomicrobiota significantly increased in the high doses GOD treatment group. *Actinobacillus* spp., associated with causing abortion, metritis, and reduced litter sizes in animals, notably decreased in the GOD group (Rycroft and Garside, 2000). The *Faecalibacterium* enriched in the G3 group is a potential novel probiotic bacterium for human diseases such as inflammatory bowel disease (IBD) (Miquel et al., 2013). *Olsenella* has a high positive relationship with obesity (Kong et al., 2019), which is reduced in the present GOD treatment groups, proving that normal or high doses of GOD addition could modulate the gut microbiota. *Enorma* is a bacterial genus often found in groups receiving long-term AGP supplements, but its abundance was reduced in the GOD-supplemented group (Dubourg et al., 2014). Chen et al. researched the fact that fermented feed groups increase the abundance of *CHCKI001* and *Faecalibacterium* in laying hens (Chen et al., 2023). The evidence confirms that the supplementation of GOD, whether at a normal or high level, effectively modulates the gut microbiota in broilers, causing no adverse effects during the farming process.

GOD-induced changes in the hindgut microbiota also led to alterations in microbial metabolic function. Jiang et al. used the same method to introduce the metabolic function of bacteria and understand the main pathway affected by probiotics (Jiang et al., 2021). Su et al. also used the metabolic function prediction to illustrate the dynamic changes of fermented feed during a two-stage solid-state fermentation process (Su et al., 2021). Enhanced carbohydrate metabolism (starch and sucrose metabolism), amino acid metabolism (amino sugar and nucleotide sugar metabolism, alanine aspartate and glutamate metabolism), glycan biosynthesis and metabolism, enzyme families (peptidases), lipid metabolism and exosome, and biosynthesis of other secondary metabolites presented that the 100 U/g GOD treatment effectively improved the metabolism function in broilers. An explanation for these metabolic function predictions is that GOD promotes probiotic proliferation and inhibits pathogens, enhancing nutrient digestibility and absorption.

A correlation analysis was conducted to identify the main gut microorganisms that GOD can use to improve gut health. Su et al. conducted a correlation analysis and discovered that the fermented feed produced butyric acid, which plays an essential role in maintaining gut immune function (Su et al., 2022). Jiang et al. utilized a specific methodological approach to demonstrate that the probiotic strain BA40 improved the growth performance of piglets, and this improvement was found to be positively correlated with the abundance of *Phascolarctobacterium*, a genus of bacteria known to be beneficial in the gastrointestinal tract (Jiang et al., 2023). Correlation analysis suggested that GOD-driven *Faecalibacterium*, *CHKCI001,* and *Mucispirllum* improved the gut health of broilers. At the genus level, *Mucispirllum* abundance was decreased significantly in response to DSS-induced colitis (Jiang et al., 2024). *Faecalibacterium* is a promising anti-inflammatory bacterium that colonizes the gut and plays an important role in the pathogenesis of IBD (Zhou et al., 2021). The correlation analysis suggested that the *Enorma*, which decreased with GOD treatment, was negatively correlated with growth performance. These findings imply that high doses of GOD can also benefit the microbial community and enhance gut health by modulating microbiota functions. Researchers utilized the Spearman correlation coefficient to explore the relationships between specific bacterial species and various outcomes. This approach helped to elucidate the complex interactions between gut microbiota and host health, providing valuable insights into the mechanisms by which GOD enhances gut health and overall performance in broilers.

## 5 Conclusion

In this study, the results of the broilers model, along with the assessment of antioxidant ability and intestinal morphology, demonstrated that GOD supplementation benefited ADG, ADFI, FCR, antioxidant function, and gut morphology. Additionally, GOD enhanced the abundance of beneficial gut probiotics, such as *Faecalibacterium*, and promoted microbial carbohydrate metabolism, particularly in starch and sucrose pathways. Correlation analysis identified *Faecalibacterium* and *CHCKI001* as two key microbial effectors contributing to the improvements in growth performance and gut health induced by GOD. These beneficial outcomes were achieved with a 100 U/g GOD supplementation, which did not exhibit any adverse effects. Notably, the high-dose GOD supplementation had a significant positive impact on broiler health and performance. Consequently, applying higher GOD doses could be advantageous in poultry and livestock farming. This foundational evidence offers valuable guidance for the practical use of high-dose GOD as an effective feed additive.

## Data Availability

The datasets presented in this study can be found in online repositories. The names of the repository/repositories and accession number(s) can be found in the article/Supplementary material.
