# Supplementary material for: Effects of high-dose glucose oxidase on broiler growth performance, antioxidant function, and intestinal microbiota in broilers
Source: Front Microbiol. 2024 Oct 28;15:1439481. doi: 10.3389/fmicb.2024.1439481 (PMC11551609; doi:10.3389/fmicb.2024.1439481)
Supplement: Supplementary file 1 [file Table_1.DOCX]

**Supplementary Table 1. Diet composition**

| **Ingredient/kg** | **Starter inclusion**  **(d1-21)** | **Grower inclusion**  **(d22-56)** | **Finisher inclusion**  **(d57-119)** |
| --- | --- | --- | --- |
| Corn | 572.5 | 625.7 | 640.0 |
| SBM | 367.5 | 315.0 | 261.0 |
| Rice bran | 0 | 0 | 20.0 |
| Soybean oil | 19.6 | 27.3 | 43.0 |
| Salt | 3.0 | 3.0 | 3.0 |
| Calcium hydroxide | 15.7 | 13.9 | 13.1 |
| Talcum powder | 15.8 | 11.9 | 16.9 |
| DL-methionine | 1.3 | 0.77 | 0.5 |
| L-lysine | 2.0 | 0 | 0 |
| Choline | 1.0 | 1.0 | 1.0 |
| Phytase^1^ | 0.1 | 0.1 | 0.1 |
| Vitamins conc^2^ | 0.3 | 0.25 | 0.3 |
| Trace minerals^3^ | 0.3 | 0.3 | 0.3 |
| Antioxidant | 0.5 | 0.4 | 0.4 |
| Coccidiostat | 0.5 | 0.5 | 0.5 |
| **Total** | **1000** | **1000** | **1000** |

^1^Phytase: 10000 U/g

**^2^**Vitamin premix provided the following per kilogram diet: vitamin A, 12,000,000 IU; vitamin D, 5,000,000 IU; vitamin E, 75 mg; vitamin K, 3 mg; cyanocobalamin,0.016 mg; folic acid, 2 mg; riboflavin, 8 mg; pyridoxine, 5 mg; biotin, 0.25 mg; thiamine, 3 mg; nicotinic acid, 55 mg; pantothenic acid, 13 mg and antioxidant ethoxyquin,50 mg.

**^3^**Mineral premix provided the following per kilogram diet: Cu sulfate, 16 mg; Mn sulfate, 60 mg; Mn oxide, 60 mg; I (iodide), 0.125 mg; Se (selenite), 0.3 mg; Fe sulfate,40 mg; Zn oxide and sulfate, 100 mg.

**Supplementary Table 2. Calculated nutrients (as-fed basis, %)**

| **Calculated nutrients, %** | **Starter inclusion**  **(d1-21)** | **Grower inclusion**  **(d22-56)** | **Finisher inclusion**  **(d57-119)** |
| --- | --- | --- | --- |
| Crude Protein≥ | 21.0 | 18.0 | 17.0 |
| Lys≥ | 1.00 | 0.90 | 0.80 |
| Met | 0.4-0.9 | 0.3-0.9 | 0.3-0.9 |
| Calcium | 0.60-1.20 | 0.6-1.2 | 0.6-1.2 |
| Phosphorus≥ | 0.50 | 0.45 | 0.40 |
| Crude Fiber≤ | 6.0 | 7.0 | 7.0 |
| NaCl | 0.30-0.80 | 0.30-0.80 | 0.30-0.80 |
| Crude Ash≤ | 8.0 | 8.0 | 8.0 |
| Water≤ | 14.0 | 14.0 | 14.0 |

**Supplementary Table 3. Primers used for qPCR**

| **Gene** | **Sequences (5’→3’)** | **Accession number** | **Length of product** |
| --- | --- | --- | --- |
| *Claudin-1* | F: AAAGGACAAAACCGTGTGGG  R: ATATTTAAGGACCGCCCTCTCC | [NM_001244539.1](https://www.ncbi.nlm.nih.gov/entrez/viewer.fcgi?db=nucleotide&id=347300347) | [264](https://www.ncbi.nlm.nih.gov/entrez/viewer.fcgi?db=nucleotide&id=347300347) |
| *Claudin-2* | F: AGAGCTCTTCGAAAGGACGG  R: ATGGATGTGCCTAACAGCCC | [NM_001410421.1](https://www.ncbi.nlm.nih.gov/entrez/viewer.fcgi?db=nucleotide&id=2283599739) | [288](https://www.ncbi.nlm.nih.gov/entrez/viewer.fcgi?db=nucleotide&id=2283599739) |
| *ZO-1* | F: GAAGTTACGTGCGGGAGCAG  R: GGGACAAAAGTCCGGGAAGC | [NM_001163574.2](https://www.ncbi.nlm.nih.gov/entrez/viewer.fcgi?db=nucleotide&id=2451183762) | [175](https://www.ncbi.nlm.nih.gov/entrez/viewer.fcgi?db=nucleotide&id=2451183762) |
| *GAPDH* | F: GGTCGGTGTGAACGGATTTG  R: AAGATGGTGATGGGCTTCCC | [NM_008084.4](https://www.ncbi.nlm.nih.gov/entrez/viewer.fcgi?db=nucleotide&id=2295558031) | [216](https://www.ncbi.nlm.nih.gov/entrez/viewer.fcgi?db=nucleotide&id=2295558031) |
